# A Model to Assess the Impact of Digital Technologies on the Health-related Quality of Life

**DOI:** 10.1017/S0266462322003245

**Published:** 2022-11-11

**Authors:** Jannik Lockl, Doreen Schick, Jens-Christian Stoetzer, Katrin Huff

**Affiliations:** 1 University of Bayreuth; 2 Branch Business & Information Systems Engineering of the Fraunhofer FIT; 3 FIM Research Center

**Keywords:** digital technologies, HRQoL, model development, impact assessment

## Abstract

**Objectives:**

Health-related quality of life (HRQoL) is a vital instrument to account for individuals’ well-being in various settings. However, no model of HRQoL allows examining the effect of digital technology on HRQoL. Therefore, we extend an established HRQoL model by adding a digital technology-related construct. We refer to this extension as the TA-HRQoL.

**Methods:**

We investigate the extended TA-HRQoL model through a survey. In the survey, we exemplify the use of digital technology through a device for self-managing bladder dysfunction. Hence, we explore whether the model extension proposed is valid and how determinants of the HRQoL affect patients with bladder dysfunction.

**Results:**

The results indicate that the use of digital technology improves the HRQoL. In our exemplary use scenario, the digital technology decreases bladder-related functional impairments and increases well-being and life satisfaction directly.

**Conclusion:**

Our study may provide evidence for the influence of digital technologies on the HRQoL, thus supporting our model extension. We consider our proposed TA-HRQoL model as valid and as useful to account for the influence of digital technology on an individual’s HRQoL. With the TA-HRQoL model, the impact of a digital technology on an individual’s HRQoL can be assessed.

## Introduction and Theoretical Background

Regardless of an individual’s personal circumstances, quality of life is an essential goal for which individuals strive (1). The interplay of various aspects influences the individual quality of life. In health care, an established concept taking the different aspects into account is the so-called health-related quality of life (HRQoL). HRQoL encompasses health-related aspects of quality of life and is regarded widely as a suitable model to assess an individual’s quality of life. Though, HRQoL can be impaired by several factors. One of these factors is suffering from a chronic disease, such as a bladder dysfunction. Individuals with a bladder dysfunction do not feel their bladder anymore or just in parts. As a result, they run the risk of losing urine at an unscheduled point of time or, even worse, to over-distend their bladder. The latter can ultimately lead to kidney insufficiencies. Maintaining independence, enabling self-management, and adapting to the disease is crucial to counteract the negative effects of a chronic disease (2). For example, patients with a bladder dysfunction avoid social events since they must face the sigma incontinence in a non-private environment. Those who can participate in social activities although suffering from the chronic disease report an improvement in their overall health status (3).

By offering interventions (e.g., using mobile devices), digital technology can be applied to support facing and improving these chronic conditions and thus help improve individuals’ health conditions (2). Examples of such interventions are mental health apps helping patients cope with milder psychological disorders (e.g., deprexis) or diet apps to change eating and moving behaviors toward a healthier lifestyle and ultimately lose weight (e.g., oviva).

However, the impact of such digital technologies is often difficult to measure, particularly regarding intangible outcomes, such as HRQoL. Following this difficulty, technology assessment in health care often does not account for such perspectives yet, although studies demonstrate the influence of technology on the quality of life (3,4). As a result, research calls for a stronger inclusion of these intangible and humanistic outcomes when investigating the effects of digital technologies on individuals (5).

Early research demonstrates how to make use of HRQoL models in technology assessment (4). However, to the best of our knowledge, we are unaware of any HRQoL model that particularly accounts for the influence of digital technologies in health care. Hence, we posit the following research question:

### How can the HRQoL-model be extended to assess the influence of digital technologies within health care?

To close this research gap and to extend the body of knowledge on the inclusion and assessment of digital technologies in HRQoL research, we extend the revised HRQoL model of Ferrans et al. (6) by adding a ‘digital technology’ construct. We place the digital technology construct within the category of environmental characteristics. Consequently, we extended the HRQoL model and refer to it as the technology-affected health-related quality of life (TA-HRQoL) model. The TA-HRQoL model includes the technological environment alongside the social and physical environment. Research acknowledges that digital technology affects social and physical aspects of the environment (e.g., 7). For example, the use of artificial intelligence (AI) in health care poses new ethical questions that were not addressed yet. If AI is used to support decisions in health care settings it is not defined yet, how doctors or other health care practitioners should handle the suggestions (e.g., diagnosis, triage category) by the AI and who is responsible in the end: The algorithm or the professional applying the AI’s decision. Moreover, also it must be discussed if the application of AI algorithms is ethically correct when taking such important decision as found in the health care sector. This all shows that digital technologies hence pose new challenges to society and research should consider these as a new aspect of individuals’ environments. Though in existing models, digital technologies only indirectly affect an individual’s HRQoL. Digital technologies are not treated as a separate construct. By adjusting the HRQoL model and adding the digital technology construct as a separate construct from the characteristics of the environment, we aim to measure the direct influence of digital technology on HRQoL and thus account for the specific impact and characteristics inherent to digital technologies.

To assess the applicability and validity of this new TA-HRQoL model, we empirically tested it among patients with bladder dysfunction. For this purpose, we developed a survey in which we exemplified the use of digital technology through the digital technology *inContAlert*. Patients suffering from bladder dysfunction were asked about the possible influence of inContAlert on their HRQoL. Patients suffering from bladder dysfunction serve as a suitable group to test the model. They all have a reduced HRQoL due to the same health issue – a missing sensation of their bladder – and there is currently no solution at hand that could solve this problem. inContAlert is an AI-enabled digital technology that allows individuals suffering from incontinence to monitor the filling status of their urinary bladder and proactively give notice when to best empty the bladder. We investigated our collected data by applying partial least squares structural equation modeling (PLS-SEM) to empirically assess and validate the relationships proposed for our structural model (8) Klicken oder tippen Sie hier, um Text einzugeben..

## Method

### Measurements and Procedure

Before conducting our research, the ethical board of the University of Bayreuth authorized the study proposal. To test our TA-HRQoL model, we conducted an online survey assessing all constructs of our model and complementary information such as participants’ demographics. We operationalized the constructs by collecting indicators from pre-validated questionnaires, if available. For constructs that so far do not have pre-validated items developed, we derived items from existing literature on HRQoL generally and specifically on HRQoL of patients with bladder dysfunction. Finally, we selected the indicators according to the best-fit principle and adapted them as well as the declarative information preceding the indicators to the study context (i.e., patients suffering from bladder dysfunction) if necessary^1^.

The initial questionnaire consisted of reflective and formative measurement models (each reflective model was also assessed with a formative one), and single-item measures. As recommended by Hair et al. (8), we conducted a pretest to test them in terms of reliability and validity. None of the 42 pre-test responses indicated suspicious response patterns or issues due to missing data. The results of the reliability and validity analysis led to the deletion of all formative measurements. We analyzed the convergent validity of the reflective measurement models. Thereby, we investigated the outer loadings and trimmed down the questionnaire according to established procedures (8). Following their approach, we retained each indicator with outer loadings of ≥ .700, whereas indicators with outer loadings of < .400 were deleted. An outer value ≥ .700 indicates that the construct explains half or more of the item’s variance, thus, it is widely regarded as a suitable threshold. Items with outer loadings <.400 are considered to have only little explanatory power, thus, we removed them. Indicators with outer loading < 700 and ≥ 400 were further investigated. Table 1 reports the validity and reliability on construct level in our pre-test.

Finally, the questionnaire was composed of reflective measurement models for the constructs Symptoms due to Bladder Dysfunction (SYMP; 9), Functional Impairments due to Bladder Dysfunction (FUNC; e.g., 10), General Health Perceptions (HEAL; 11), Overall Quality of Life (QUAL; e.g., 12), Depression (DEPR; 13), Digital Technology Support for Bladder Management (TECH; 14), Social Support (SOCI; 15), and Physical Environment (PHYS; 16). The constructs Main Type of Bladder Dysfunction (TYPE; derived from 17) and Bladder Management Method (MANA; 9) were assessed by single-item measurements. All items were translated into German, making it possible for respondents to complete the questionnaire either in English or in German. To ensure linguistic correctness of the translation we applied the commonly used forward-backward translation method. In accordance with this method, we translated each item into German and let other proficient English speakers translate the German version back into English to elaborate if the retranslation was the same and hence the meaning of the item was kept withing the German version. Table 2 shows the exemplary items for the construct TECH.

At the start of the survey participant information provided potential respondents with details concerning the goal of the survey, its procedure, and the privacy policy. After agreeing to participate in the survey, the privacy policy and the status of the respondent (patient or assistant of such), participants completed the survey.

### Sample

A prerequisite to participate in our study was suffering from any kind of bladder dysfunction oneself (patient questionnaire) or knowing and supporting someone with bladder dysfunction (assistant questionnaire). Participants had to be at least 18 years old and have sufficient knowledge of either English or German language. To obtain a diverse and extensive sample we applied convenience sampling. The survey was distributed in a four-month period (18^th^ November 2020 to 15^th^ February 2021) in associations, social media groups, forums, and information portals addressing bladder dysfunction or diseases commonly associated it.

To determine the minimum required sample size, we followed the recommendations of Hair et al (8). According to Hair et al. (8) a statistical power of 80 percent is a commonly used value, thus the recommendations indicated a minimum sample size of *N* = 129 required for a statistical power of 80 percent (R^2^ ≥ .100 or α = .05) (8).

A total of 536 responses were gathered during the survey period. Responses were excluded from further analysis if 1) no consent to participate in the study was given 2) the respondent was neither a patient suffering bladder dysfunction nor an assistant of such, 3) the questionnaire was not fully answered, or 4) if the responses showed missing data and suspicious response patterns. More detailed, if the missing data for a specific participant exceeded 15 percent of the survey, the response was excluded from further analysis as well as suspicious patterns such as straight-lining, diagonal lining, and alternating extreme pole responses. Of the 536 responses collected, 353 were complete. We removed four responses due to suspicious response patterns and missing data. The final set of 349 questionnaires consists of 337 (96.56 percent) patient and 12 (3.43 percent) assistant responses. The assistant questionnaire did collect demographic information of the person they assisted. On average, the respondents were 45.12 years old (min = 18; max = 81; SD = 12.76). 217 (62.18 percent) of the respondents were female, 131 (37.54 percent) male, and 1 (0.29 percent) reported "Others" as their gender. 153 (43.84 percent) of the respondents were married, and 132 (37.82 percent) reported as single. 255 (73.06 percent) of them held at least a high school degree and 86 (24.64 percent) currently unable to work or unemployed. 144 (41.26 percent) regulated their bladder function by urinating in the toilet. The remaining 205 (58.74 percent) applied another bladder management method. Lastly, almost half of the respondents displayed a sensory and motoric bladder dysfunction simultaneously.

## Results

To examine our research question, we extended the TA-HRQoL model by applying PLS-SEM (18) using the statistical software SmartPLS3.

### Measurement Models

Prior to the evaluation of the structural model, we assessed the measurement models. We refrained from evaluating the single-item measures in terms of their reliability and validity. Only five indicators of the reflective measurement models displayed outer loadings smaller than .700. We examined the impact of omitting the items on the composite reliability. Based on the results, we dropped three items. The AVE values of our constructs ranged from .575 to .818, which indicates convergent validity at the construct level. HTMT values indicate the discriminant validity of a constructs. All HTMT values of the reflective measurement models had desirable values (values ranged from .049 to .806), indicating discriminant validity. For all measurement models, the values of Cronbach’s alpha and composite reliability were in the range of .600 and .950, implying internal consistency reliability. Lastly, we examined our model for collinearity issues by calculating the VIF. All VIF values were below the recommended threshold of five. Thus, collinearity among predictor constructs was not problematic.

### Structural Model

**Figure 1:**
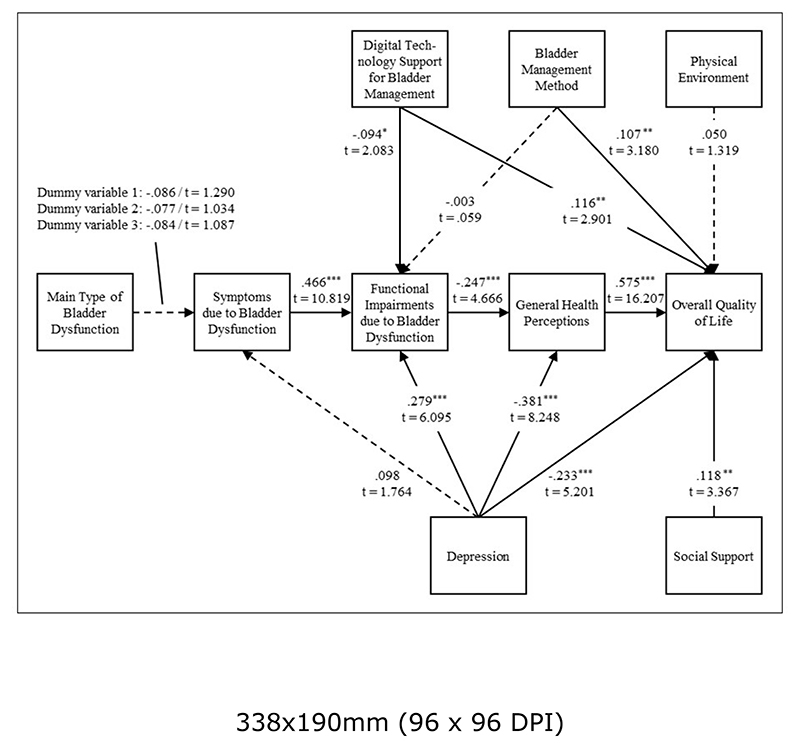
Research model with path coefficients, t values, and significance levels (* significant at p < .050; ** significant at p < .010; *** significant at p < .001) Source: Own illustration; Data: Own survey (data set: full sample).

Table 3 shows the strength of each path coefficient and their reflective significance for each hypothesized relationship in the structural model. Figure 1 presents the research model, including the strength and significance of the path coefficients. Following established procedures, we used p-values to assess the significance of our path coefficients. P-values allow us to report the statistical probability of inappropriately disapproving a true null hypothesis.

Neither the construct *TYPE* nor the construct *DEPR* share a significant relationship with *SYMP*. However, *SYMP* and *DEPR* have a strong and highly significant positive effect on *FUNC*. The construct *TECH*, on the other hand, has a significant negative impact on *FUNC*, while *MANA* has no significant effect on *FUNC*. Besides, the negative effects of *FUNC* and *DEPR* on *HEAL* are significant. Furthermore, *HEAL* has the strongest significant relationship with *QUAL*. Notably, *HEAL, MANA, TECH*, and *SOCI* share a significant positive relationship with *QUAL*, while the relationship of *DEPR* with *QUAL* is significant but negative. In contrast, *PHYS* does not have a significant relationship with *QUAL*

Table 4 shows that seven constructs have a significant total effect on the construct Overall Quality of Life. Thereby, General Health Perceptions (.575) and Digital Technology Support for Bladder Management (.129) have the strongest positive total effect, whereas Depression (-.498) and Functional Impairments due to Bladder Dysfunction (-.142) have the strongest negative total effect.

The path model explained R^2^ = .610 of the variance in the dependent variable Overall Quality of Life. The R^2^ of the structural model for Functional Impairments due to Bladder Dysfunction (.335) and General Health Perceptions (.267) is weak. Furthermore, the model does not have explanatory power (.014) for Symptoms due to Bladder Dysfunction.

Overall, these significant paths and the R^2^ values show that our enhanced TA-HRQoL model and the relationships between the different constructs are shown in our data, thus giving supporting evidence for our model.

Moreover, except for the relationship between Digital Technology Support for Bladder Management and Functional Impairments due to Bladder Dysfunction, all significant structural paths display at least small effect sizes. Consequently, omitting the construct Digital Technology Support for Bladder Management from the research model would not alter the R^2^ value of Functional Impairments due to Bladder Dysfunction substantially. Our data, thus, indicates that Digital Technology Support for Bladder Management does not have a substantive impact on Functional Impairments due to Bladder Dysfunction. Bladder Management Method (.028), Depression (.090), Digital Technology Support for Bladder Management (.034), and Social Support (.029) have a small effect on Overall Quality of Life. Likewise, Functional Impairments due to Bladder Dysfunction (.075) evince a small effect on General Health Perceptions. Moreover, Depression has a small effect on Functional Impairments due to Bladder Dysfunction (.115) and a medium effect on General Health Perceptions (.178). Besides, Symptoms due to Bladder Dysfunction has a medium effect size of .320 on the Functional Impairments due to Bladder Dysfunction. Lastly, the construct General Health Perceptions shows a large effect of .648 on Overall Quality of Life. For all remaining construct combinations, the independent variables have no effect on their dependent variables. The effect sizes show that the relationships between the different constructs are substantial with two relationships even having medium effect sizes and on relationship showing even a large effect size.

In sum, the results demonstrate that the data provides evidence for the applicability of our model in further research.

## Discussions and Conclusion

The aim of our study was to assess the influence of digital technology on the HRQoL. To analyze that influence, we propose the TA-HRQoL model, which we evaluated through an online survey and structural equation modeling. Overall, the results of our analysis showed that the elaborated TA-HRQoL model seems to be a valid model with almost all paths showing significant effects (except the paths TYPE → SYMP, MANA → FUNC, DEPR → SYMP, and PHYS → QUAL). Especially the relationships between the constructs Digital Technology Support for Bladder Management and Functional Impairments due to Bladder Dysfunction as well as Digital Technology Support for Bladder Management and Overall Quality of Life were of interest to our research question to explain the effects of digital technologies on HRQoL. Our data revealed significant positive relationships for both hypotheses, thus indicating a substantial influence of digital technologies on HRQoL.

### Theoretical and Practical Contribution

Our results show that by reducing the bladder-related functional impairments, digital technology indirectly affects the HRQoL through the mediation of health perceptions. Support through digital technology reduces the negative impact of functional restrictions on an individual’s general health perception. When general health perceptions improve, in turn, a patient’s HRQoL is affected positively. Consequently, the results of our survey provide empirical evidence for the relations hypothesized within our study and, consequently, for the validity of our TA-HRQoL model. The TA-HRQoL model allows to assess the influence of a digital technology on an individual’s HRQoL and its reflective determinants. The exemplary digital technology used in our study allows patients to track their own body functions, here the state of bladder level. Many other diseases also need a constant surveillance of body functions. To just name one, e. g. patients with diabetes need to constantly check their blood sugar levels to avoid negative consequences of the disease. Digital technologies, such as inContAlert, enable these tracking functions within health care. Thus, we consider the TA-HRQoL to also be applicable to digital technologies used within the contexts of other diseases. Our TA-HRQoL model contributes to the ongoing discussion about the impact of technologies in the health care domain (19). Our study shows that it is important to consider the influence of digital technologies when assessing HRQoL. To the best of our knowledge, no previous study proposed digital technology as an individual construct to explain the dependent variables of HRQoL. Our result – digital technology significantly influences the HRQoL – supports earlier research (e.g., 20, 21), which suggested that digital technology can positively affect HRQoL. Thus, we contribute to research on technology assessment in health care and validate existing research in this domain (4). Our study is a starting point to assess the impact of digital technologies on humanistic outcomes, such as HRQoL.

However, it must be considered that certain disparities concerning the availability, knowledge, and use of digital technologies have an influence on how patients use them. These disparities also indirectly affect the patients’ HRQoL in the context of the use of digital technology. Consequently, the consideration of such inequalities among the target group is critical to the design and development of digital technology-based interventions (22). Since our study demonstrated the positive effect of technology on the HRQoL, we recommend governments and health insurers to guarantee access to adequate health technology. Digital technology that succeeds in improving a patient’s health status can ultimately reduce the cost of health care by lowering the need for traditional treatments in the future (23).

Also, the TA-HRQoL model sets the scene to assess the medical and economic value of digital technologies in health care. So far, an indicator to evaluate health services or programs is the health-adjusted expectancy of life (HALE). HALE is a measurement that includes life expectancy, mortality, and quality of life (24). More precisely, it is the life expectancy adjusted for HRQoL (25). Our validated TA-HRQoL model could integrate the influence of digital technology into the HALE model. Hence, we posit that digital technologies also have the potential to improve life expectancy.

Thus, we contribute to research on HRQoL as well as on the impact and effects of digital technologies in health care. Research calls to address the missing link between the impact of digital technologies and so-called humanistic outcomes that are intangible results of the use of digital technologies (5). We address this call for future research by demonstrating the potential of digital technologies to improve humanistic outcomes of their use for individuals (5).

### Limitations and Future Research

Even though we highlight the theoretical contribution of our work, we acknowledge its limitations. First, our survey design indeed included an introduction of the digital technology, but it was rather brief. Respondents may not have understood all aspects of the technology correctly, although we applied control questions to ensure a basic level of understanding. Second, expectations of patients regarding the usability of the technology and its integration into daily routines may have differed from experiences they would gain from actual use. Hence, researchers ought to repeat the study once the technology is freely available for use. Third, even though we applied all standard procedures to ensure the validity and reliability of our findings, we cannot entirely rule out empirical biases (8).

These limitations in mind our study provides various fruitful avenues for further research on TA-HRQoL. To overcome the empirical limitations, we suggest the use of a longitudinal study design and multiple methods for data collection (e.g., surveys, experiments, medical records, patient examination). Doing so allows for (dis-)confirming and expanding the insights we obtained based on the expected use of digital technologies. Further, the TA-HRQoL model should also be tested in various study settings and among different patient groups. Research in this area has the potential to unfold an understanding of the relationship between digital technology and a patient’s quality of life. Our study thereby expands the body of knowledge on the influence of technology on HRQoL, while the results are valuable for academia and practice, such as for physicians and health care providers, simultaneously. Finally, future research could use our TA-HRQoL model to evaluate the impact of digital technologies on life expectancy, e.g., using HALE as a measurement.

## Conclusion

In health care, digital technology offers a wide range of application scenarios. Targeting self-management of chronic disease patients, the use of digital technology aims to increase their independence, their physical ability, and their social inclusion. The use of digital technology could affect the HRQoL of such patients considerably. However, no HRQoL model accounts for the influence of digital technology. In the study at hand, we extended an established HRQoL model by adding a digital technology construct. We refer to this refined model as the TA-HRQoL model. Our study demonstrates that digital technology has significant positive influence on the HRQoL. The results allow us to confirm the hypothesis that digital technology affects HRQoL. The TA-HRQoL model enables the assessment of the influence of a digital technology. The TA-HRQoL, thus, contributes to the body of knowledge of technology assessment and the field of health care management in general.

## Figures and Tables

**Table 1:** Convergent validity and internal consistency reliability based on data obtained from the pretest

Construct	Average variance extracted	Composite reliability	Cronbach’s alpha
DEPR	.667	.909	.875
FUNC	.641	.934	.920
HEAL	.659	.906	.869
PHYS	.638	.840	.724
QUAL	.858	.948	.917
SOCI	.641	.926	.907
SYMP	.534	.886	.846
TECH	.783	.956	.946

**Table 2:** Exemplary items for construct *Digital Technology Support for Bladder Management*

Item No.	Item	Source
TECH1	By using inContAlert for bladder management, I would need less time to selfmanage my bladder dysfunction.	14
TECH2	Using inContAlert would improve the self-management of my bladder dysfunction.	
TECH3	Using inContAlert would allow me to improve my use of aids for bladder management (e.g., diapers, pads, catheters).	
TECH4	Using inContAlert would help me to have better control over my bladder dysfunction.	
TECH5	Using inContAlert would make it easier to self-manage my bladder dysfunction.	
TECH6	I would find inContAlert useful to self-management my bladder dysfunction.	

**Table 3:** Results of the hypotheses testing

Hypothesis		Path coefficient	Support	f^2^	Effect size
H1	TYPE → SYMP	-.086		.005	
		-.077	No	.003	–
		-.084		.004	
H2	SYMP → FUNC	.466^***^	Yes	.320	Medium
H3	FUNC → HEAL	-.247^***^	Yes	.075	Small
H4	HEAL → QUAL	.575^***^	Yes	.648	Large
H5	MANA → FUNC	-.003	No	.000	–
H6	MANA → QUAL	.107^**^	Yes	.028	Small
H7	DEPR → SYMP	.098	No	.010	–
H8	DEPR → FUNC	.279^***^	Yes	.115	Small
H9	DEPR → HEAL	-.381^***^	Yes	.178	Medium
H10	DEPR → QUAL	-.233^***^	Yes	.090	Small
H11	TECH → FUNC	-.094^*^	Yes	.013	–
H12	TECH → QUAL	.116^**^	Yes	.034	Small
H13	SOCI → QUAL	.118^**^	Yes	.029	Small
H14	PHYS → QUAL	.050	No	.006	–

Source: Own illustration; Data: Own survey (data set: full sample).

* significant at p < .050;

** significant at p < .010;

*** significant at p < .001

**Table 4:** Total effects on *Overall Quality of Life*

Rank	Construct	Total effect
1	HEAL	.575^***^
2	DEPR	-.498^***^
3	FUNC	-.142^***^
4	TECH	.129^**^
5	SOCI	.118^**^
6	MANA	.108^**^
7	SYMP	-.066^***^
8	PHYS	.050
9	TYPE	.006
		.005
		.006

Source: Own illustration; Data: Own survey (data set: full sample).

* significant at p < .050;

** significant at p < .010;

*** significant at p < .001

## Footnotes

1 The different scales and items had the following specifications: SOCI1-9, TECH1-6 and FORM1 from 1 (*strongly disagree*) to 5 (*strongly agree*). FORM3 from 1 (*minimal*) to 4 (*large*). DEPR1-4 from 1 (*as much as ever*) to 5 (*not at all*). DEPR5-7 and FUNC7-10 from 1 (*never*) to 5 (*always*). PHYS1 from 1 (*not at all*) to 5 (*extremely*). PHYS2-4 and FORM2 from 1 (*very dissatisfied*) to 5 (*very satisfied*). SYMP1 from 1 (*Zero – I [She/he] would not have urine leakage*) to 5 (*more than once a day*). SYMP2-3 from 1 (*Zero – I [She/he] would not have urine leakage*) to 5 (*large (clothes/pads are soaked)*). SYMP4 from 1 (*never*) to 4 (*many times a day*). SYMP 5 from 1 (*I don’t [She/he doesn’t] think about urinating. I have [She/he has] a catheter or stoma bag*) to 4 (*I [She/he] would have to do this right away or I [she/he] may leak urine*). SYMP6 from 1 (*never*) to 5 (*three or more times*). SYMP7 from 1 (*more than 3 hours*) to 4 (*less than an hour*). SYMP8 from 1 (*This would not be an issue for me [her/him]. I [She/he] would not have urine leakage*) to 5 (*less than an hour*). SYMP9 from 1 (*never*) to 4 (*most of the time*). SYMP10 from 1 (*This isn’t an issue for me [her/him]. I don’t [She/he doesn’t] feel my [her/his] bladder, or I use [she/he uses] a catheter or stoma bag*) to 4 (*Agree. This would happen most of the time*). SYMP11 from 1 (*This isn’t an issue for me [her/him]. I use [She/he uses] a catheter or stoma bag*) to 4 (*Would drop out*). SYMP12 from 1 (*This isn’t an issue for me [her/him]. I use [She/he uses] a catheter or stoma bag*) to 4 (*Agree. This would happen most of the time*). SYMP13 from 1 (*never*) to 5 (*once a month, or more*). FUNC1-6 and FORM4 from 1 (*not at all*) to 5 (*extremely*). HEAL1 and QUAL2-3 from 1 (*very poor*) to 5 (*very good*). HEAL2-5 from 1 (*definitely true*) to 5 (*definitely false*). QUAL1 from 1 (*Most people would have a better life than I do [she/he does].*) to 5 (*My [Her/his] life would be better than anyone else’s*.). QUAL4 was implemented as a slider from 1 (*good*) to 5 (*bad*) and QUAL5 used a scale from 1 (*terrible*) to 5 (*delighted*).
